# Work Environment Characteristics and Emotional Intelligence as Correlates of Nurses’ Compassion Satisfaction and Compassion Fatigue: A Cross-Sectional Survey Study

**DOI:** 10.3390/nursrep11040079

**Published:** 2021-10-26

**Authors:** Stephanie Maillet, Emily Read

**Affiliations:** 1 Faculté d’Administration, Campus de Moncton, Université de Moncton, Moncton, NB E1A 3E9, Canada; 2 Faculty of Nursing, University of New Brunswick, Moncton, NB E1C 0L2, Canada; eread@unb.ca

**Keywords:** compassion fatigue, compassion satisfaction, job strain, psychological demands, decision latitude, social support, emotional intelligence, nursing

## Abstract

This cross-sectional survey study examined the relationship between Canadian nurses’ work environment characteristics, emotional intelligence, compassion fatigue and compassion satisfaction (*n* = 1271). Psychological demands, decision latitude, supervisor and coworker support, and emotional intelligence (EI) were significantly correlated with nurses’ compassion satisfaction and compassion fatigue, except for two EI subscales. Furthermore, these relationships were stronger for compassion satisfaction than compassion fatigue, suggesting that they are influenced by different factors. Our results highlight the importance of creating reasonable psychological demands, empowering nurses to make decisions in their jobs, supportive relationships at work, and fostering the development of nurses’ EI.

## 1. Introduction

Nurses engage in profound human interactions with patients and families who seek support, healing, and encouragement during times of suffering [1,2]. Nurses do this compassionately, expending the emotional energy required to sustain caring, empathetic, and meaningful relationships [3,4]. Nurses practice with kindness and empathy towards their patients and often find pleasure from caregiving, which fosters a continued desire to contribute to the provision of patient care and stewardship to the profession [5,6]. The term “compassion satisfaction” has been used to describe the professional satisfaction derived from caregiving and stems from personal fulfillment found in helping others cope with stressful situations [7,8]. Compassion satisfaction occurs when empathy drives altruistic behaviors on the part of the caregiver and results in the alleviation of patient suffering [6].

While the caring and compassionate connections established between nurses and patients can be deeply rewarding, the increasing complexity in healthcare, along with the intense human interactions required of nurses (i.e., emotional labor), often leads to high levels of compassion fatigue [9,10]. Considered by some as the “cost of caring” [11], compassion fatigue refers to the “disengagement of caregivers from their patients, which culminates in a reduction or inability to provide the patient care that is deemed empathetic and compassionate. It is the loss of meaningful and purposeful interaction between caregivers and patients” [12] (p. 4). Compassion fatigue is associated with a panoply of negative emotional outcomes, such as anxiety, anger, frustration, and feelings of helplessness [13,14,15]. Researchers have suggested that these negative emotions may be exacerbated due to job strain and an unsupportive work environment [16,17,18].

### 1.1. Job Strain

Job strain is a response to working environments where employees experience a combination of high psychological demands and low decision latitude [19]. Psychological demands refer to the sustained cognitive and emotional effort required to accomplish one’s work, while decision latitude represents the control that an employee has over their work and the extent to which they can be autonomous, creative, and flexible in deciding which skills and processes to use in accomplishing their day-to-day work [19,20]. In nursing, adequate psychological demands and decision latitude are significantly associated with nurses’ enacted scope of practice, including assessment and care planning, the teaching of patients and families, communication and care coordination, and knowledge updating and utilization [21,22]. Managing psychological demands and optimizing nurses’ decision latitude is necessary for nurses to enact their scope of practice, and consequently, improve their quality of work-life, as well as the quality of care and patient safety [22].

### 1.2. Job Strain and Compassion Fatigue and Compassion Satisfaction

Job strain has been studied extensively in many occupations, including nursing which is known to be a stressful career with a high level of emotional labor [23]. Research over the last several decades has consistently found that nurses often experience job strain due to intense psychological demands that include high workloads, time pressure, competing urgencies, short staffing, inadequate social support, uncertainty, and emotionally intense situations, often in combination with limited decision latitude [24,25]. In such circumstances, nurses’ scope of practice becomes suboptimal [22] and a process of energy depletion and wearing down may develop over time, leading to higher compassion fatigue and decreased compassion satisfaction [24,25,26]. Thus, we propose that both components of job strain—high psychological demands and low decision latitude—are associated with increased compassion fatigue and decreased compassion satisfaction among nurses.

### 1.3. Social Support

Workplace social support is another factor that aids in reducing the impact of psychological demands and low control on the development of job strain [19]. Different sources of social support can alleviate the effects of job strain on nurses, such as supervisor and coworker support [27,28]. According to Feeney and Collins [29] (p. 6), “one important function that social relationships serve is to support thriving through adversity, not only by buffering individuals from the negative effects of stress but also by helping them to emerge from the stressor in a way that enables them to flourish either because or despite their circumstances”. Supervisors and coworkers can engage in helpful social interactions in the workplace by offering support, help, advice and appreciation to mitigate the adverse effects of job strain [30]. For example, researchers have suggested that social support may aid in not only hindering the effects of compassion fatigue but also in promoting compassion satisfaction when continuously exposed to a stressful work environment [6,31,32]. Consistent with this theoretical perspective, we propose that when nurses feel that they are well supported by their supervisor and co-workers, they will experience lower levels of compassion fatigue and higher levels of compassion satisfaction.

### 1.4. Emotional Intelligence

In addition, we argue that these work environment characteristics are not sufficient in accounting for the prevalence of compassion satisfaction and compassion fatigue in nursing. While appropriate psychological demands, decision latitude, as well as supervisor and coworker support, seem to minimize compassion fatigue while promoting compassion satisfaction, intrapersonal resources, such as emotional intelligence have also been recognized to be critical in nurses’ ability to handle the intensely emotional situations inherent to the profession that place them at risk for compassion fatigue [10,33,34].

Emotional intelligence has been defined as the “ability to monitor one’s own and others’ feelings, to discriminate among them, and to use this information to guide one’s thinking and actions” [35] (p. 189). Individuals with a high level of emotional intelligence are generally more adept at evaluating, understanding, and managing their own emotions, and tend to perceive having more social support [36,37]. Individuals who have increased awareness, understanding and ability to manage their own emotions also perceive themselves as more compassionate [38], which is essential for working in the nursing profession [39].

Because of the relational and emotional nature of nurse-patient interactions [33], nurses are exposed to and experience a wide range of intense emotional interactions when dealing with patients and their families [40]. In general, nurses are expected to display a genuine and caring demeanor, express empathy for patients and show an understanding of their pain and suffering [41]. Nurses are required to regulate their emotions to conform to socially desirable expressions of emotion, such as empathy, caring and concern, but to avoid negative emotions, such as anger, anxiety, or frustration [40,42]. In the nursing work environment, where intense emotions are guaranteed to surface, nurses are required to not only properly perceive but also manage their own and others’ emotions to provide higher quality care to patients, but also for their own psychological wellbeing [40,43].

The relationship between emotional intelligence and both compassion fatigue and compassion satisfaction has not been extensively studied in nursing. In a study conducted by Dafeeah, Etlohami, & Ghulou [44], results revealed that higher emotional intelligence was associated with increased compassionate attitudes towards patients. A more recent study conducted in 2017 by Beauvais, Andreychik, & Henkel [33] found that compassion fatigue was inversely associated with emotional intelligence whereas a positive relationship was found with compassion satisfaction. Thus, higher levels of emotional intelligence could protect nurses from developing compassion fatigue while also contributing to higher levels of compassion satisfaction.

## 2. Materials and Methods

### 2.1. Aims

**Figure 1:**
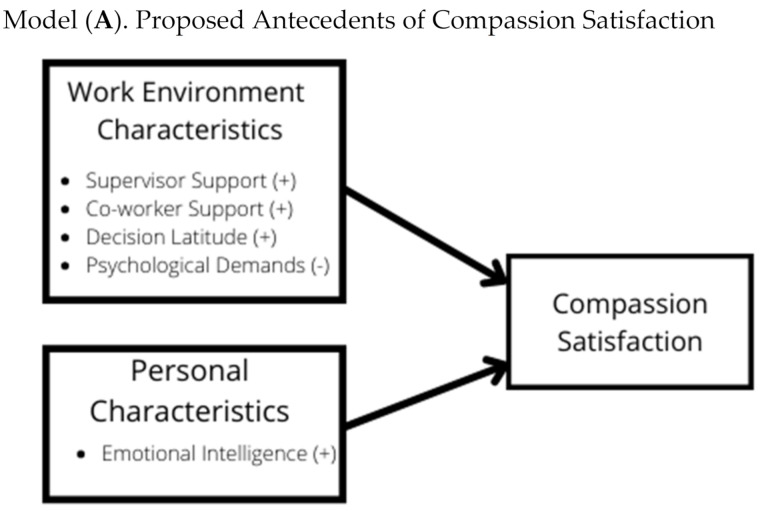

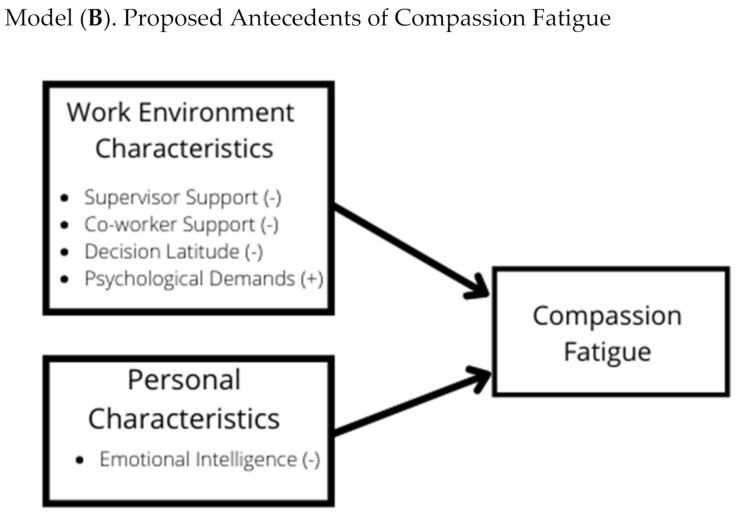
Hypothesized Models.

The current study aimed to examine two hypothesized models linking work environment characteristics (psychological demands, decision latitude, supervisor support, and coworker support) and emotional intelligence to compassion satisfaction (model A in Figure 1) and compassion fatigue (model B in Figure 1) among Registered Nurses (RNs).

### 2.2. Design

A cross-sectional online survey of Canadian RNs was conducted. RNs were invited through their provincial regulatory body and via social media to complete an online survey hosted on SurveyMonkey. Data were collected between September and December 2016. Ethical approval of the study was obtained from University Ethics Review Board prior to participant recruitment.

### 2.3. Participants

Participant characteristics are provided in Table 1. The majority of our sample (27.3%) were between ages 26–35 years of age (*n* = 347), followed by 26% between the ages of 46–55 (*n* = 330). Consistent with current statistics about the nursing workforce, almost 93% of the sample were female and the majority (60.3%) had a bachelor’s degree (*n* = 766). In terms of provincial representation, 39.6% of the sample was employed in New Brunswick (*n* = 503), 20.3% in Nova Scotia (*n* = 258), 15.5% in Manitoba (*n* = 197), 13.1% in Quebec (167), 10.5% in Alberta (133), and single digit representation from British Columbia (*n* = 3), Saskatchewan (*n* = 2), Ontario (*n* =4), and PEI (*n* = 3). Participants had a range of nursing experience, with 41.5% (*n* = 528) being within the first ten years of their career, 58.4% working an average of 31 to 40 h per week (*n* = 743) and most working in medical/surgical specialty areas.

### 2.4. Data Collection and Ethical Considerations

This study was approved by the University Institutional Review Board (no. 1516-071). Informed consent was obtained by all subjects involved in the study. Data were collected using an online survey comprised of demographic questions and previously validated self-report questionnaires. Work environment characteristics were assessed using the Psychological Demands, Decision Latitude, Supervisor Support, and Coworker Support subscales from Karasek’s Job Content Questionnaire (JCQ) [19]. Overall emotional intelligence was assessed with the Schutte Self-Report Emotional Intelligence test (SSEIT) [45]. The revised ProQOL-21 item and scoring approach [46] was used to assess compassion fatigue and compassion satisfaction. Table 2 provides an overview of the instruments, their scoring, and Cronbach’s alpha reliability from the current study.

### 2.5. Data Analysis

The Statistical Package for the Social Sciences (SPSS) (version 22.0, IBM Corporation, New York, United States, 2014) was used for data cleaning and analysis. We only removed cases with missing values if they did not respond to any of the items for one or more of the variables. To maintain the integrity of the data, we did not impute any missing values. Prior to analyzing the relationships between main study variables, the measurement model of each questionnaire was evaluated using confirmatory factor analysis (CFA) with maximum likelihood (ML) estimation in MPlus [47]. Cronbach’s alpha reliabilities were also calculated for each subscale and total scale. Pearson’s r correlation coefficients in SPSS were used to assess the significance (*p* < 0.01), magnitude, and direction (positive or negative) of the relationships between the study variables.

## 3. Results

### 3.1. Descriptive Results

As shown in Table 3, the mean score for ratings of supervisor support was 10.26 (SD = 3.10), while coworker support was 12.39 (SD = 2.11). The mean score for psychological demands was 23.69 (SD = 3.59), while the mean score for decision latitude was 72.48 (SD = 10.04). Overall EI had a mean score of 124.56 (SD = 12.16) which is lower than past studies which have reported mean scores of 130.94 (SD = 20.25) for women [45]. The subscales of EI had mean scores of 37.80 (SD = 4.88) for perceptions of emotions, 34.26 (SD = 4.76) for managing own emotions, 29.79 (SD = 3.81) for managing others’ emotions, and 22.66 (SD = 3.15) for utilizations of emotions. Mean scores for compassion satisfaction were 25.16 (SD = 6.15) while the mean for compassion fatigue was 26.75 (SD = 26.75). Based on the percentile cut points for the ProQOL-21 provided by Heritage et al. [46], this places our participants near the 50th percentile for compassion satisfaction and above the 75th percentile for compassion fatigue.

### 3.2. Correlational Results

Table 3 also shows the correlations between the main study variables. Compassion fatigue and compassion satisfaction were inversely related to one another (r = −0.48). Compassion satisfaction was significantly related to all the proposed antecedent variables. In terms of work environment characteristics, compassion satisfaction was positively correlated with supervisor support (r = 0.33), coworker support (r = 0.31), and decision latitude (r = 0.40) and negatively correlated with psychological demands (r = −0.25). Overall EI (r = 0.43) and each subscale of EI were also positively correlated with compassion satisfaction (r = 0.19–0.52).

All proposed antecedent variables were also significantly correlated with compassion fatigue except for two subscales of EI (perceptions of emotions and utilizations of emotions). Supervisor support (r = −0.30), coworker support (r = −0.26), and decision latitude (r = −0.22) were negatively correlated with compassion fatigue, while higher psychological demands were positively associated with greater levels of compassion fatigue (r = 0.22). Total EI (r = −0.20), managing one’s own emotions (−0.26), and managing others’ emotions (r = −0.14) were negatively correlated with compassion fatigue.

## 4. Discussion

To our knowledge, this is the only study to examine the relationships between job strain (psychological demands and decision latitude), social support, emotional intelligence, as well as compassion fatigue and compassion satisfaction among a large sample of RNs. This research is unique and significant because it addresses areas that have not yet been extensively studied in nursing. Further, to prevent measurement contamination in the assessment of compassion fatigue and compassion satisfaction, this study uses the revised ProQOL scale, a robust measurement alternative suggested by Heritage et al. [46]. Thus, another contribution of this research is that it is one of the first studies to use this version of the ProQOL questionnaire.

In our hypothesized models, we suggested that psychological demands would be negatively linked to compassion satisfaction and positively linked to compassion fatigue, while decision latitude, supervisor support, coworker support and emotional intelligence would be positively linked to compassion satisfaction and negatively linked to compassion fatigue. In the current study, these proposed relationships between variables were supported, which is in line with related research. For example, in a nursing study conducted by Barr [31], nurses who felt more strongly that work demands pulled them in different and sometimes opposing directions and nurses who felt more overburdened by the volume of work had higher levels of compassion fatigue. Similarly, a study among nursing midwives found that continuous exposure to distressing situations and lack of decision latitude increased susceptibility to developing compassion fatigue [48].

While nurses cannot avoid the job stain inherent to their profession, researchers have found that those who receive social support are less susceptible to compassion fatigue and are better equipped to cope with their work demands [49,50,51,52,53]. In a study conducted among 862 nurses by Pergol-Metko & Czyzewski [52], results showed that a positive perception of social support was associated with a lower level of compassion fatigue. Researchers have also found that social support was associated with higher compassion satisfaction among nurses [31,54]. In terms of supervisor support, these results could be explained by the fact that when nurses can access supervisory support through mobilization of organizational resources, direct task assistance, feedback, advice, autonomy and decision-latitude over their work, nurses’ perception of job strain and its adverse effects are mitigated [55,56]. Furthermore, because of their daily interactions and shared experiences, a positive and supportive coworker network can facilitate mutual help, guidance, assistance, and positive affect [57].

In line with our study results, emotional intelligence is another important personal resource that has been shown to be critical for nurses to cope effectively with job strain and compassion fatigue in the context of contemporary nursing [39,58,59]. For instance, Araque [58] and Kaur et al. [59] found evidence that managing emotions are highly related to caring behaviors among nurses. Indeed, emotional intelligence appears to be a key factor underlying a nurses’ ability to provide compassionate care to patients [38,60]. Furthermore, it seems that individuals with higher emotional intelligence are better able to express their emotions in a socially desirable way, while those who have lower emotional intelligence may have more difficulty doing so [61].

In the current study, while all proposed relationships between variables were corroborated, two subscales of emotional intelligence were not significantly related to compassion fatigue: perception of emotions, which describes the extent to which an individual can perceive, appraise, and express emotions; and utilization of emotions, which refers to the extent to which people report being able to utilize emotions in problem-solving and decision making [45,61]. We argue that it may not be the perception and utilization of emotion that lead to compassion fatigue, but rather incongruence between experienced emotions and outward displays of emotion [62]. When there is a mismatch between actual feelings and displayed emotions, nurses could become indifferent to their job and less empathic in their interactions with patients [63,64]. To provide compassionate care to patients despite the emotions they are experiencing, or lack thereof, nurses are expected to display a caring and empathetic demeanor toward their patients, and to either change or suppress their actual feelings to show the expected emotions [41]. However, this inauthentic emotional labor may create emotional dissonance, thereby further increasing compassion fatigue [62,65]. On the contrary, when nurses display naturally and genuinely felt emotions, an intentional effort to feel certain emotions is not required before expression, which promotes emotional compatibility [66,67] and could consequently foster compassion satisfaction.

To better understand these emotional phenomena, future research efforts could aim to replicate the current study by extending beyond emotional intelligence and focusing on the impact of emotional display through deep or surface acting [62]. Surface acting involves regulation of emotional expression and suppression of one’s felt emotions, whereas deep acting involves attempting to change one’s felt emotions to meet the role demands [63].

Some drawbacks of the research should be acknowledged. An important limitation of this study is related to the cross-sectional correlational research design, which prevented us from drawing conclusions about causality and directionality. In addition, the convenience sample may result in data that are not representative of the entire population. Another important limitation is related to common method bias due to the use of self-report questionnaires.

## 5. Conclusions

Our findings add to growing evidence that supporting and developing nurses needs to be a priority for the nursing profession and healthcare organizations. Preventing compassion fatigue and fostering compassion satisfaction can be accomplished by providing nurses with a work environment that does not burden them with unnecessary psychological demands, fosters RN autonomy within their scope of practice (decision latitude) and provides them with tangible support from both supervisors and coworkers. Due to limited generalizability of our study results, nursing work environment assessments of current job demands, job resources, emotional intelligence, compassion fatigue and compassion satisfaction using established assessment tools could offer insights and help to monitor nurses’ intervention needs. These targeted assessments could occur on different levels, whether individual, departmental, and organizational, to investigate the prevalence of compassion fatigue and compassion satisfaction and the relative importance of their antecedents. Such assessments are highly relevant given the ever-increasing pressure and challenges in nursing work due to the ongoing worldwide pandemic, coupled with a nursing shortage and difficulty in retaining qualified nursing staff [27,68]. Our findings also suggest that compassion fatigue and compassion satisfaction are not influenced by the same factors and that further investigation into these phenomena using both qualitative and quantitative research approaches are warranted.

## Figures and Tables

**Table 1:** Participant Characteristics (*n* = 1271).

Variable	Categories	*n*	%
Age	18–25	129	10.1
	26–35	347	27.3
	36–45	246	19.4
	46–55	330	26.0
	56 and older	200	15.7
	Not reported	19	1.5
Gender	Male	81	6.4
	Female	1180	92.8
	Not reported	10	0.8
Marital Status	Single	206	16.2
	Married	644	50.7
	Divorced/Separated	226	17.8
	Common law	174	13.7
	Widowed	14	1.1
	Not reported	7	0.6
Highest level of education	Diploma/Certificate	412	32.4
	Baccalaureate	766	60.3
	Masters	75	5.9
	Doctorate	3	0.2
	Other	4	0.3
	Not reported	11	0.9
Province of Employment	British Columbia	3	0.2
	Alberta	133	10.5
	Saskatchewan	2	0.2
	Manitoba	197	15.5
	Ontario	4	0.3
	Quebec	167	13.1
	New Brunswick	503	39.6
	Nova Scotia	258	20.3
	PEI	3	0.2
	Not Reported	1	0.1
Specialty Area	Medical/Surgical	444	34.9
	Maternal/Child	117	9.2
	Mental health/Psychiatric	105	8.6
	Critical Care/ER	238	18.7
	Community	136	10.7
	Geriatrics	146	11.5
	Rehabilitation	31	2.4
	Not reported	54	4.2
Years as a nurse	0–10	528	41.5
	11–20	219	17.2
	21–30	267	21
	31–40	216	17
	41–50	30	2.4
	51–60	1	0.1
	Not reported	10	0.8
Hours worked per week	0–10	13	1.0
	11–20	48	3.8
	21–30	224	17.6
	31–40	743	58.4
	41–50	181	14.3
	51–60	23	1.8
	60 plus	17	1.3
	Not reported	22	1.7

**Table 2:** Study Instruments.

Variable	Instrument	Scoring	# of Items	Score Range	Cronbach’s α (Current Study)
Psychological demands	Job Content Questionnaire (Karasek et al., 1998).	Items are rated on a Likert scale from 1 = Strongly Disagree to 4 = Strongly Agree Psychological demands = [(Q19 + Q20) × 3 + (15 − (Q22 + Q23 + Q26)) × 2]	5	12–48	0.74
Decision Latitude	Job Content Questionnaire (Karasek et al., 1998).	Items are rated on a Likert scale from 1 = Strongly Disagree to 4 = Strongly Agree Decision latitude = skill discretion + decision-making authority), where: Skill discretion = [q1 + q3 + q5 + q7 + q9 + (5 − q2)] × 2 Decision-making authority = [2 × (q4 + q6 + q8)] × 2	9	24–96	0.69
Supervisor support	Job Content Questionnaire (Karasek et al., 1998)	Items are rated on a Likert scale from 1 = Strongly Disagree to 4 = Strongly Agree	4	4–16	0.92
Coworker support	Job Content Questionnaire (Karasek et al., 1998)	Items are rated on a Likert scale from 1 = Strongly Disagree to 4 = Strongly Agree	4	4–16	0.78
Emotional Intelligence	Schutte Self-Report Emotional Intelligence Test (Schutte et al., 1998)	Items are rated on a Likert scale from 1 = Strongly Disagree to 5 = Strongly Agree	33	33–165	0.85
		Total and subscale scores are calculated by taking the sum of all items.			
		*Perceptions of Emotions*	10		0.81
		*Managing Own Emotions*	9		0.83
		*Managing others’ emotions*	8		0.66
		*Utilizations of emotions*	6		0.67
Compassion satisfaction	ProQoL-21 (Heritage et al., 2018)	Items are rated on a Likert scale from 1 = never to 5 = very often Total score calculated by recoding and taking the sum of 10 items.	10	10–50	0.92
Compassion Fatigue	ProQoL-21 (Heritage et al., 2018)	Items are rated on a Likert scale from 1 = never to 5 = very often Total score calculated by recoding and taking the sum of 11 items.	11	11–55	0.94

**Table 3:** Means, Standard Deviations, and Pearson Correlations of Proposed Antecedents of Compassion Fatigue and Compassion Satisfaction.

				Pearson’s r	
Variable	M	SD	Range	Compassion Fatigue	Compassion Satisfaction
Supervisor Support	10.36	3.10	2–16	−0.30 *	0.33 *
Coworker Support	12.39	2.11	2–16	−0.26 *	0.31 *
Psych job demands	23.69	3.59	8–34	0.22 *	−0.25 *
Decision latitude	72.48	10.04	32–96	−0.22 *	0.40 *
Total Emotional Intelligence	124.56	13.16	36–163	−0.20 *	0.43 *
*Perceptions of Emotions*	37.80	4.88	9–50	−0.05	0.19 *
*Managing Own Emotions*	34.26	4.76	14–45	−0.36 *	0.52 *
*Managing others’ emotions*	29.79	3.81	8–40	−0.14 *	0.37 *
*Utilizations of emotions*	22.66	3.15	4–30	−0.06	0.26 *
Compassion Fatigue	26.75	7.10	11–46	−	−0.48 *
Compassion Satisfaction	25.16	6.15	10–36	−0.48 *	−

Notes: * indicates that the correlation is significant, *p* < 0.05; Out of range lower-bound scores are due to non-response.

## Data Availability

Participants of this study did not agree for their data to be shared publicly, so supporting data is not available.
